# Whole-genome sequencing of *Klebsiella pneumoniae* isolated from clinical specimens in Chattogram, Bangladesh

**DOI:** 10.1128/mra.00442-24

**Published:** 2024-06-28

**Authors:** Afroza Akter Tanni, Farjana Sharmen, Kallyan Chakma, Farhana Yasmin, Al-Shahriar Akash, Md. Ashikur Alim Akash, Sumaiya Hafiz Riana, Sajia Afrin, Jannatul Ferdous, Nahid Sultana, Sanjoy Kanti Biswas, S. M. Rafiqul Islam, Adnan Mannan

**Affiliations:** 1 Department of Genetic Engineering and Biotechnology, University of Chittagong, Chattogram, Bangladesh; 2 Next-generation Sequencing, Research and Innovation Laboratory Chittagong (NRICh), Biotechnology Research and Innovation Centre (BRIC), University of Chittagong, Chattogram, Bangladesh; 3 Department of Microbiology, Chattogram Maa O Shishu Hospital, Agrabad, Chattogram, Bangladesh; Rochester Institute of Technology, Rochester, New York, USA

**Keywords:** genome, *Klebsiella pneumonia*, multidrug-resistant, Bangladesh, Chattogram

## Abstract

The emergence of multidrug-resistant *Klebsiella pneumoniae* (Kpn) is a global concern due to the increasing rate of mortality and hospital cost burden in the affected population. This study reports the whole-genome sequences of nine multidrug-resistant Kpn from a hospital in Chattogram city of Bangladesh.

## ANNOUNCEMENT

*Klebsiella pneumoniae* (Kpn) is an opportunistic bacterium of the Enterobacteriaceae family that has been reported as a critically prioritized infectious agent by the World Health Organization (1). The emergence of carbapenem-resistant serotypes in the species is a great concern because of their diverse range of infections that result in higher mortality rate (2, 3). According to a study, Kpn is a predominant cause for various human-associated infections (4). Sequence surveillance is advised to comprehend the rapid evolution of antibiotic-resistant Kpn strains consisting of a variety of transposable elements (4, 5).

Kpn isolates were obtained from clinical specimens between August 2022 and October 2022, from Chattogram Maa O Shishu Hospital Medical College (CMOSHMC), Bangladesh (ethical approval reference: CMOSHMC/IRB/2021/026). The antimicrobial susceptibility profile was obtained by the Kirbey–Bauer method according to the guidelines of the Clinical and Laboratory Standard Institute (CLSI) (6). The antimicrobial susceptibility profile is summarized in Table 1. Primarily, clinical isolates were cultured on MacConkey agar media plates and incubated at 37°C for 18–22 h to obtain pure *Klebsiella* colonies. The colony morphology was observed the next day, followed by biochemical tests [e.g., IMViC tests and Triple sugar iron (TSI) test] for initial detection of Kpn (7, 8). Genomic DNA was extracted from pure colonies by following the manufacturer’s instruction of the DNA extraction kit (QIAmp DNA extraction kit, Qiagen, Germany) and extracted DNA quality was screened with a Nanodrop spectrophotometer (Thermo Fisher Scientific, USA) (9). The pair-end library was prepared by using a NEBNext Ultra II FS DNA library prep kit (Illumina, San Diego, CA) following manufacturer guideline, and library size was selected with AMPure XP beads (BECKMAN COULTER) (10, 11). The quality of genomic library was screened by agarose gel electrophoresis, and concentration was measured with Qubit fluorometer (Thermo Fisher Scientific). Finally, sequencing was run on Illumina iSeq100 platform by a paired-end 300-cycle protocol (12).

**TABLE 1:** Antibiotic susceptibility profiles, assembly data, and annotations of reported Kpn genomes^*a*^

Features		Kpn002	Kpn003	Kpn005	Kpn006	Kpn007	Kpn010	Kpn011	Kpn012	Kpn016
Sample source	Type of patient	Neonate	Neonate	Neonate	Neonate	Old Adult	Neonate	Young Adult	Neonate	Old Adult
	Specimens	Blood	Blood	Tracheal Aspirate	Blood	Sputum	Pus	Urine	Tracheal Aspirate	Sputum
Resistance profile	Isolates resistant to	CTR, CFM, AMK, GEN, CFX, CPM, CAZ	CTR, GEN, AMK, CPM, TG, CAZ	CIP, CTR, CFM, GEN, AMK, CFX, LVX, CAZ	CIP, CTR, CFM, GEN, AMK, CFX, LVX, CAZ	CTR, CFM, GEN, AMK, CFX, CPM, CAZ	CFM, GEN, AMK, CPM, CAZ	CTR, GEN, AMK, CPM, TG, CAZ	CFM, GEN, AMK, CFX, TG, CPM, LVX, CAZ	GEN, AMK, CFM, CPM, CAZ
	AMR Associated genes	Sul1, KPC-2, NDM-5, SHV-11	Sul1, KPC-2, NDM-5, SHV-11	Sul1, KPC-2, NDM-5, SHV-11	Sul, NDM-5, SHV-11	SHV-75	Sul1, KPC-2, NDM-5, SHV-11	NDM-5 SHV-11	Sul1, NDM-1, SHV-28	SHV-27
Genome statistics	Total length	5,337,186	5,339,452	5,338,711	5,659,151	5,442,638	5,336,009	4,779,154	5,510461	5,565,244
	Genome coverage	61	48	61	38	86	65	10	12	84
	Guanine-Cytosine (GC) (%)	57.3	57.3	57.3	57.1	57.2	57.3	53.2	56.3	57.1
	Contigs	59	57	73	77	63	64	1121	798	93
	N50 values	350,709	308,627	270,158	310,209	209,534	326,664	6,179	13,103	240,835
										
Genome annotation data	Genes (total)	5,316	5,318	5,318	5,674	5,308	5,313	5,406	5,945	5,533
	CDSs (total)	5,225	5,224	5,226	5,585	5,228	5,224	5,358	5,873	5,444
	Genes (coding)	5,146	5,145	5,150	5,456	5,098	5,144	5,155	5,691	5,313
	CDSs (with protein)	5,146	5,145	5,150	5,456	5,098	5,144	5,155	5,691	5,313
	Genes (RNA)	91	94	92	89	80	89	48	72	89
	complete rRNAs	1 (5S)	1 (5S)	1 (5S)	2, 1, 1 (5S, 16S, 23S)	1, 1 (5S, 16S)	1 (5S)	1, 1 (16S, 23S)	1, 1, 1 (5S, 16S, 23S)	1 (5S)
	tRNAs	77	79	77	74	68	75	39	64	75
	ncRNAs	10	11	11	11	9	10	6	5	10
	Pseudo genes (total)	79	79	76	129	130	80	203	182	131

^*a*^ Kpn = *Klebsiella pneumoniae*; CIP, Ciprofloxacin; GEN, Gentamycin; CTR, Ceftriaxone; CFM, Cefixime; AMK, Amikacin; CFX, Cefuroxime; TG, Tigecycline; CPM, Cefepime; LVX, Levofloxacin; CAZ, Ceftazidime; CDS = coding sequences; ncRNA = non-coding RNA.

Low-quality sequence reads and adapter sequences were eliminated using the Trimmomatic v 0.40 tool (13). The read quality was checked in FASTQC v.0.11.9, and the paired-end reads were merged and assembled using SPAdes 3.14.1 (14, 15). Then, QUAST v5.2 was used to determine the statistics of assembled genomes (16). The annotation process was carried out using the NCBI Prokaryotic Genome Annotation Pipeline (PGAP) v6.1 (17, 18), and the resulting data are summarized in Table 1. The Kpn strains were taxonomically detected from Kraken2 (19, 20), and then, Kleborate tool was used to detect and analyze genomes of Kpn and associated species complex (21). The default settings were applied to all software.

Based on the genomic analysis conducted via Kleborate tool, the reported Kpn isolates belong to the following sequence types (STs)- ST11, ST15, ST16, ST420, ST86, and ST277. These identified STs of the study concur with previously published reports that indicated a prevalence of ST15 and ST23 in South Asian hospital setting (22) alongside the circulation of hypervirulent ST11 in different regions of China (23). Further, both genome analysis and genomic data can be utilized to create a pipeline for genomic surveillance for antimicrobial resistance in Bangladesh.

## Data Availability

The WGS data of nine Kpn strains has been deposited in GenBank under accession number JBBMLY000000000, JBBMLX000000000, JBBMLW000000000, JBBMLV000000000, JBBMLU000000000, JBBMLT000000000, JBBMLS000000000, JBBMLR000000000, JBBMLQ000000000. The Illumina Sequence reads are available in the NCBI Sequence Reads Archive (SRA) under accession number SRR25920021, SRR25920020, SRR25920013, SRR25920012, SRR25920011, SRR25920008, SRR25920007, SRR25920019 and SRR25920015.
